# Highly-Efficient Sulfonated UiO-66(Zr) Optical Fiber for Rapid Detection of Trace Levels of Pb^2+^

**DOI:** 10.3390/ijms22116053

**Published:** 2021-06-03

**Authors:** Marziyeh Nazari, Abbas Amini, Nathan T. Eden, Mikel C. Duke, Chun Cheng, Matthew R. Hill

**Affiliations:** 1 Mathematics and Physics Department, School of Engineering, Australian College of Kuwait, Safat 13015, Kuwait; m.nazari@ack.edu.kw; 2 Institute for Sustainable Industries and Livable Cities (ISILC), Victoria University, Melbourne, VIC 8001, Australia; mikel.duke@vu.edu.au; 3 Mechanical Engineering Department, School of Engineering, Australian College of Kuwait, Safat 13015, Kuwait; 4 Center for Infrastructure Engineering, Western Sydney University, Penrith, NSW 2751, Australia; 5 Department of Chemical Engineering, Monash University, Clayton, VIC 3800, Australia; nathan.eden@monash.edu (N.T.E.); matthew.hill@csiro.au (M.R.H.); 6 Department of Materials Science and Engineering, Southern University of Science and Technology (SUSTech), Shenzhen 518055, China; chengc@sustech.edu.cn; 7 CSIRO Manufacturing, Clayton, VIC 3168, Australia

**Keywords:** nano-bio detectors and sensors, nano-bio systems, aqueous quality, nanobiotechnology, optical fiber vesicle, sulfonated MOFs

## Abstract

Lead detection for biological environments, aqueous resources, and medicinal compounds, rely mainly on either utilizing bulky lab equipment such as ICP-OES or ready-made sensors, which are based on colorimetry with some limitations including selectivity and low interference. Remote, rapid and efficient detection of heavy metals in aqueous solutions at ppm and sub-ppm levels have faced significant challenges that requires novel compounds with such ability. Here, a UiO-66(Zr) metal-organic framework (MOF) functionalized with SO${}_{3}$H group (SO${}_{3}$H-UiO-66(Zr)) is deposited on the end-face of an optical fiber to detect lead cations (Pb${}^{2+}$) in water at 25.2, 43.5 and 64.0 ppm levels. The SO${}_{3}$H-UiO-66(Zr) system provides a Fabry–Perot sensor by which the lead ions are detected rapidly (milliseconds) at 25.2 ppm aqueous solution reflecting in the wavelength shifts in interference spectrum. The proposed removal mechanism is based on the adsorption of [Pb(OH${}_{2}$)${}_{6}$]${}^{2+}$ in water on SO${}_{3}$H-UiO-66(Zr) due to a strong affinity between functionalized MOF and lead. This is the first work that advances a multi-purpose optical fiber-coated functional MOF as an on-site remote chemical sensor for rapid detection of lead cations at extremely low concentrations in an aqueous system.

## 1. Introduction

Lead (Pb${}^{2+}$) and other small-scale substances (e.g., soot aerosol, ammonia, and arsenic) [1,2] are known as deadly widespread toxic pollutants in the environment at macro- to nano-scale due to recent industrialization and agricultural activities [3,4]. A serious concern has raised for Canadian [5], the U.S. [6], and old European mega-cities [7] with high amounts of lead nano-substances found in drinking water which are originated from old pipes or chemical reactions occurred in corroded plumbing components [8].

According to American Academy of Family Physicians (AAFP), any level of detectable lead in human blood is abnormal [9]. Recurring exposure to low levels of Pb creates serious health issues for infants and children such as slow development and permanent intellectual disability [10,11]. In addition to various sources, lead can be taken up by fishes and other aquatic organisms from water accumulating in humans tissues after consumption [12], and then resulting in neurological [13], hematopoietic [14], musculoskeletal [15], cardiac function [16] and reproductive damages [17,18]. Despite the detrimental properties of lead to living objects, there is yet a significant gap to efficiently and abruptly detect and characterize lead at extremely small levels in bioactive compounds [19,20].

Methodologically, atomic absorption spectroscopy (AAS), atomic emission spectroscopy (AES), X-ray fluorescence (XRF), and inductively coupled plasma-optical emission spectrometry (ICP-OES) are the commonly used laboratory techniques for measuring lead contents in drinking water [21]. To use the above expensive and complicated equipment, water samples should be collected on-site, transported to a laboratory, and tested by trained professionals. This process for a large-scale determination of lead concentration is costly, time-consuming, and effortful. As yet, there are considerable efforts to develop sensors to allow discrete measurements of lead contents at-the-source for home-users. The existing detection mechanisms are based on colorimetry [22,23], biosensing [24], and electrochemical configurations [25], which, in addition to their low detection limits, have many other constraints. For instance, matrix interferences in the colorimetric method either disrupt the reaction between the reagent and the analyte or interfere the spectrometric light measurement [21]. In the biosensing method, more complex biological molecules, e.g., Daphnia magna, are needed with higher selectivity and less interference for reagents [26]. In the electrochemical sensing technique, lead-selective membranes are utilized on electrodes, where the response can be impacted by the interference from other ions presented in the water sample, effecting the solution ionic strength and potential drift [21]. Thus, an accessible, fast, sustainable, and efficient technology can fill this gap to detect Pb${}^{2+}$ in aqueous resources at low ppm and ppb levels [27].

So far, there are increasing interests in recent years to take advantage of advanced materials to adsorb Pb${}^{2+}$ nanoparticles at deficit concentrations from aqueous resources [28,29,30,31,32,33]. Metal-organic frameworks (MOFs) are highly porous 3D-materials made of metal ions linked with organic ligands. The size and shape of pores are affected by the coordination geometry of metals (e.g., tetrahedral, octahedral) that dictates the number of bounded ligands. Proper selection of metal ions and ligands can yield crystals with ultrahigh porosity as well as high thermal and chemical stability. Among MOFs, UiO-66(Zr) or Zr${}_{6}$O${}_{4}$(OH)${}_{4}$ has a stable crystalline structure in water, introducing it as a promising candidate for sensing the aqueous contaminants and purification purposes [34]. Our recent studies have shown the favorable capability of UiO-66 for removing rhodamine-B [35], methyl viologen [36], and 4-aminopyridine [37] from water contents. The functionalization of UiO-66(Zr) with proper chemical groups is suggested to enhance its low affinity with Pb${}^{2+}$ ions.

In this work, a new setup is introduced via functionalization of UiO-66(Zr) with SO${}_{3}$H to capture Pb${}^{2+}$ at low ppm levels in aqueous environments. Due to the strong coordination of Pb${}^{2+}$ with SO${}_{3}^{-}$ group, a small quantity of lead (<0.5 ppm) was left in the solution. To create a remote sensing setup for rapid detection within the range of a few milliseconds, the functionalized MOF was coated at the end-face of an optical fiber (single-mode fiber, SMF-28) and used as an in-fiber Fabry–Perot interferometer (FPI) [38]. The changes to the MOF optical properties due to adsorption of Pb${}^{2+}$ were detected via wavelength shifts in the interference spectrum.

## 2. Methods

SO${}_{3}$H-UiO-66(Zr) sensing element was synthesized through a growth solution proposed by Okoro et al. [24] with some modifications. Briefly, ZrCl${}_{4}$ (1.93 g, 8.3 mmol) and monosodium 2-sulfoterephthalate (NaSO${}_{3}$-BDC, 2.2 g, 8.2 mmol) were dissolved under stirring with *N*,*N*-dimethylformamide (DMF, 100 mL) and concentrated HCl (37%, 1.3 mL). Then, glacial acetic acid (100%, 16.6 mL; 35 equiv.) was added as a modulator. The mixture was continuously stirred for 2 h and left for 24 h at 120 °C in a pre-heated oven. After naturally cooling down to room temperature, the SO${}_{3}$H-UiO-66(Zr) nanoparticles were centrifuged at 20,000 rpm for 10 min and washed with fresh DMF (at least three times), then with pure methanol (at least three times), and kept under constant stirring with dichloromethane (DCM) overnight. They were dried for 5 h at 60 °C under reduced pressure.

The SMF-28 optical fiber was made of molten silica glass heated up to 2200 °C and drawn into tubes with varied diameters. In this work, fibers with a cladding diameter of 125 ± 0.7 m and a core diameter of 8.2 m were utilized. Before coating, the optical fiber made of SiO${}_{2}$ glass was treated with hydroxyl (OH) functional groups. A piranha solution was prepared from a 3:1 mixture of sulfuric acid (98%) and hydrogen peroxide (30%), into which optical fibers were cleaved at a right angle and incubated for 30 min. To grow the MOF sensing element on the exposed surface of the optical fibers, the OH-functionalized optical fibers were placed in an untreated precursor solution of SO${}_{3}$H-UiO-66(Zr), heated at 120 °C for 24 h. After this procedure, the fibers were gently washed with DMF, methanol and DCM, to remove unreacted reagents.

Fabry–Perot interferometry (FPI) was used as the optical detection method. When the light is propagated down to the core of fiber, it interacts with the sensing element. The element is in contact with lead-contaminated water, and by capturing the Pb${}^{2+}$, its optical thickness and refractive index change. The reflected light sends this information to the detector, where a custom-written software (MATLAB [39]) processes it [35,36,37]. The resultant FPI spectrum (interferograms) is obtained in correlation with the lead concentration in distilled (DI) water.

To test the lead uptake capacity of as-synthesized MOF, different amounts of SO${}_{3}$H-UiO-66(Zr) (10, 15, 20 and 25 mg) were separately added to 5 mL diluted solution with 25.2 (0.12 mM), 43.5 (0.21 mM) and 64.0 (0.31 mM) ppm lead under constant stirring for 10, 30 and 60 min (impregnation). Before taking a sample (aliquot) from the middle of the vial, the solid was separated by centrifuging at 20,000 rpm for 5 min, then resting for 4 h at room temperature. The adsorption mechanism of Pb${}^{2+}$ onto SO${}_{3}$H-UiO-66(Zr) was characterized by X-ray diffraction (XRD), N${}_{2}$ gas porosimetry, and Fourier-transform infrared spectroscopy (FT-IR) methods (Appendix A).

## 3. Results and Discussion

### 3.1. Pb${}^{2+}$ Uptake by SO${}_{3}$H-UiO-66(Zr) Powder

**Figure 1:**
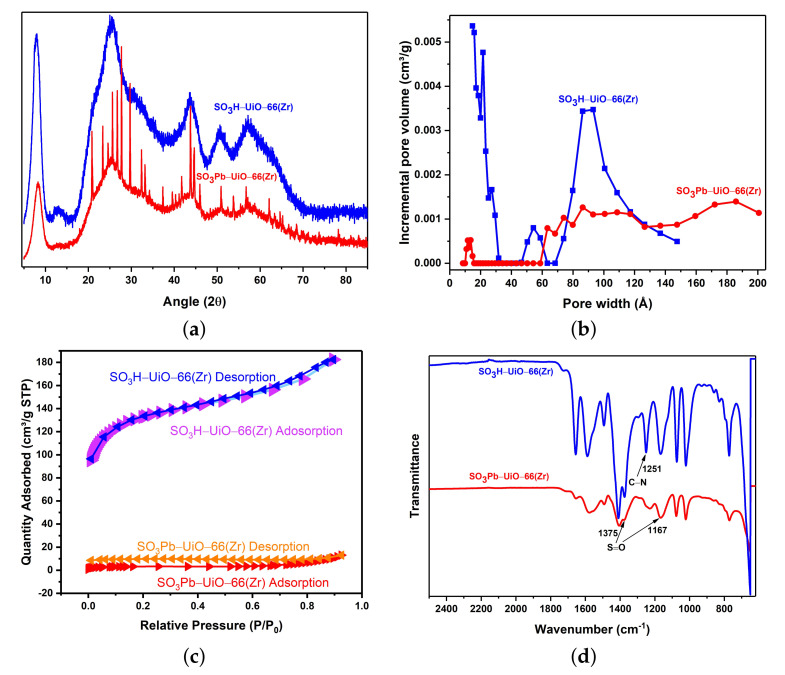
(**a**) XRD curves, (**b**) Pore size distribution, (**c**) N${}_{2}$ adsorption/desorption isotherms, and (**d**) FT-IR spectra of SO${}_{3}$H-UiO-66(Zr) and SO${}_{3}$Pb-UiO-66(Zr) powder.

**Figure A1:**
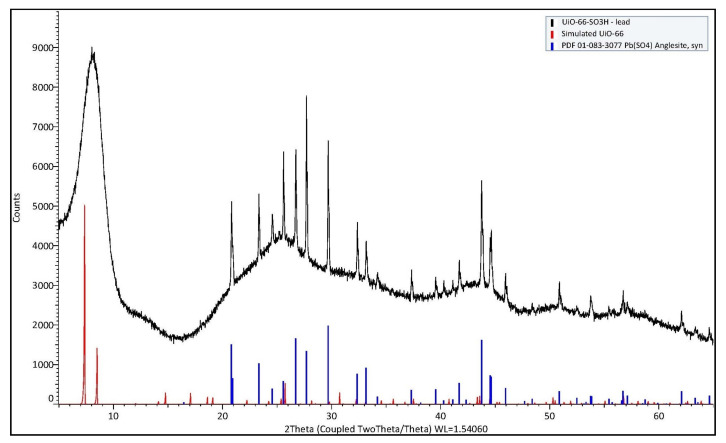
Diffractogram pattern of SO${}_{3}$Pb-UiO-66(Zr) at 2θ region (5°–65°), superimposed over the simulated diffractograms of UiO-66 and PbSO${}_{4}$ anglesite.

Figure 1a displays the XRD pattern of SO${}_{3}$H-UiO-66(Zr) and SO${}_{3}$Pb-UiO-66(Zr) powder. A change in the XRD pattern of the amorphous SO${}_{3}$H-UiO-66(Zr) sample occurrs by appearing the additional peaks in the SO${}_{3}$Pb-UiO-66(Zr) sample (prescribed to PbSO${}_{4}$ post-adsorption through peak matching in crystallographic database (Figure A1)). This suggests the formation of a material that matches with the crystallographic geometry of PbSO${}_{4}$. The cleavage energy of the sulfonate group is prohibitively high under the adsorption conditions (pH = 5.6, room temperature/pressure, and in aqueous environment), and Pb(NO${}_{3}$)${}_{2}$ is soluble in water with different crystallographic geometries. The pH was achieved by atmospheric carbon dioxide dissolving into the DI water as a natural process. To avoid addition of interferent compounds to the water/Pb context, the pH was not regulated. Therefore, we conclude that Pb${}^{2+}$ cation is absorbed onto the sulfonate groups of SO${}_{3}$H-UiO-66(Zr) structure where the tetrahedral R-SO${}_{3}$ of the 2-sulfoterephthalate linker replaces the tetrahedral SO${}_{4}$ of PbSO${}_{4}$.

Standard N${}_{2}$ gas porosimetry measurements confirm the uptake of lead within SO${}_{3}$H-UiO-66(Zr), where the Brunauer-Emmett-Teller (BET) surface area decreases from 491 m${}^{2}$/g to 12 m${}^{2}$/g upon the lead uptake. Figure 1b shows the pore size distribution of SO${}_{3}$H-UiO-66(Zr) before/after the lead uptake; this is associated with the additional mass of Pb${}^{2+}$ incorporated into the compound as well as the reduction of internal pore volume. The adsorption/desorption isotherms of SO${}_{3}$H-UiO-66(Zr) before/after the lead uptake are shown in Figure 1c.

Upon the uptake of Pb${}^{2+}$, significant changes in the observed transmittance reflect the change of dipole moment due to the adsorbed Pb${}^{2+}$. Unchanged peak positions associated with the UiO-66(Zr) framework indicate that the structure does not undergo significant changes while the crystallinity increases (Figure 1d). For instance, the characteristic S=O stretching at 1375 cm${}^{-1}$ and 1167 cm${}^{-1}$ become weaker in the SO${}_{3}$Pb-UiO-66(Zr) sample, while C−H stretching peak at 1251 cm${}^{-1}$ has a very low intensity. Defects in SO${}_{3}$H-UiO-66(Zr) reduces the density of functional groups that interact with the laser. The reduced transmittance is seen in the figure in the gesture of smaller peaks.

### 3.2. Pb${}^{2+}$ Uptake of SO${}_{3}$H-UiO-66(Zr) Powder

Inductively coupled plasma-optical emission spectrometry (ICP-OES) was conducted to determine the trace level of lead in the aliquots after its impregnation. By nonlinear regression modelling, the equilibrium/optimized level of lead uptake capacity of as-synthesized MOF was determined as 33.7 mg with a maximum 94% uptake (r-squared fit of 99.7%).

**Figure 2:**
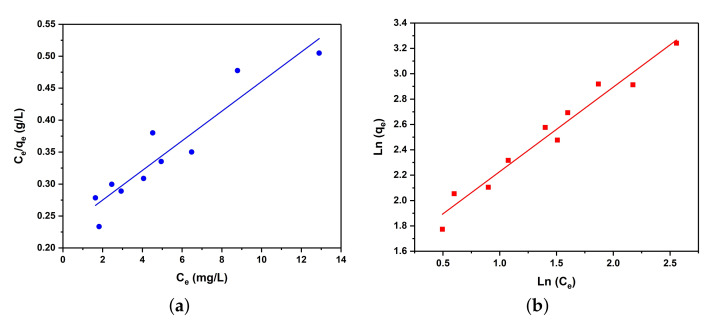
Adsorption isotherms fitted by linearized form of (**a**) Langmuir and (**b**) Freundlich model for adsorbed Pb${}^{2+}$ by SO${}_{3}$H-UiO-66(Zr)。

Langmuir and Freundlich models are used to investigate the adsorbent/adsorbate interaction in MOFs. Langmuir isotherm assumes that in the uptake process, the adsorbent places itself as a monolayer on the surface of the material. This model can be linearly expressed in the form of Equation (1):

(1) $$C_{e}/q_{e}=K_{L}/q_{m}+C_{e}/q_{m}$$

where *C*${}_{e}$ is the equilibrium solution concentration (mg/L or ppm), $q_{e}$ is the amount of Pb${}^{2+}$ at the equilibrium (mg/g), $K_{L}$ is the Langmuir adsorption constant related to the energy of adsorption, and $q_{m}$ is the maximum adsorption capacity of the MOF (mg/g). A plot of $C_{e}$/$q_{e}$ (y-axis) vs. $C_{e}$ (x-axis) allows the calculation of $q_{m}$ and $K_{L}$ parameters (Figure 2a). Another model, empirical Freundlich isotherm, assumes that the distribution of active sites in the MOF is homogeneous and is linearly expressed in the form of Equation (2):

(2) $$\ln q_{e}=\ln K_{f}+\ln C_{e}/n$$

here, $C_{e}$ is the equilibrium solution concentration (mg/L or ppm), $q_{e}$ is the amount of Pb${}^{2+}$ at the equilibrium (mg/g), $K_{f}$ is the Freundlich adsorption constant, and *n* is an empirical value. A plot of $\ln q_{e}$ (y-axis) vs. $\ln C_{e}$ (x-axis) determines the magnitudes of $K_{f}$ and *n* (Figure 2b).

Regression analysis of the data, the average values of K${}_{f}$, K${}_{L}$, *q*${}_{m}$ and *n* are shown in Table 1. In this study, the Freundlich model shows a better predictor than the Langmuir model with slightly higher R${}^{2}$, suggesting that the uptake of lead cations occurrs mainly and homogeneously throughout the entire of MOF framework. Moreover, the calculated average *n* value for the adsorption of Pb${}^{2+}$ is 1.66, showing a good efficiency of SO${}_{3}$Pb-UiO-66(Zr) for the lead adsorption [40,41].

**Figure A2:**
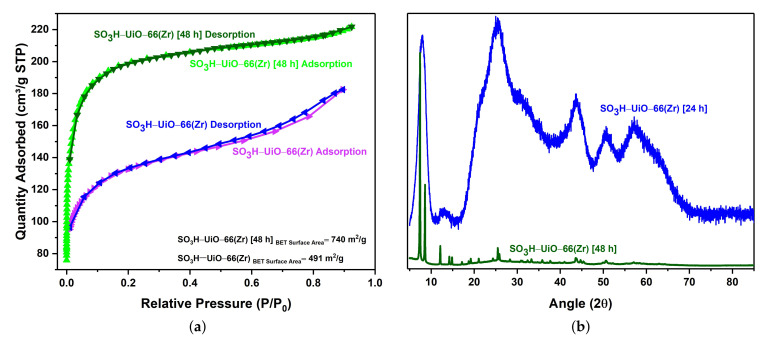
(**a**) N${}_{2}$ adsorption/desorption isotherms and (**b**) XRD curves of SO${}_{3}$H-UiO-66(Zr) [24 h] and SO${}_{3}$H-UiO-66(Zr) [48 h] powder.

To determine whether a high degree of crystallinity affected the lead uptake, the untreated precursor solution of SO${}_{3}$H-UiO-66(Zr) was heated at 120 °C for 48 h (rather 24 h); a highly crystallized as-synthesized MOF was achieved. As seen in the XRD patterns (Figure A2b), more defined Bragg peaks were observed for the MOF heated for 48 h in comparison to the one heated for 24 h. Based on the ICP-OES results, 10 mg of this highly formed MOF led to <0.5 ppm (2.4 M) left-over lead in the initial 25.2 ppm-solution (99.99% uptake) for all impregnation ranges (10, 30 and 60 min). In contrast, non-functionalized UiO-66(Zr) MOF reduced the lead content from 25.2 ppm to 23.1 ppm after 60 min constant stirring.

### 3.3. Optical Fiber Sensing

**Figure 3:**
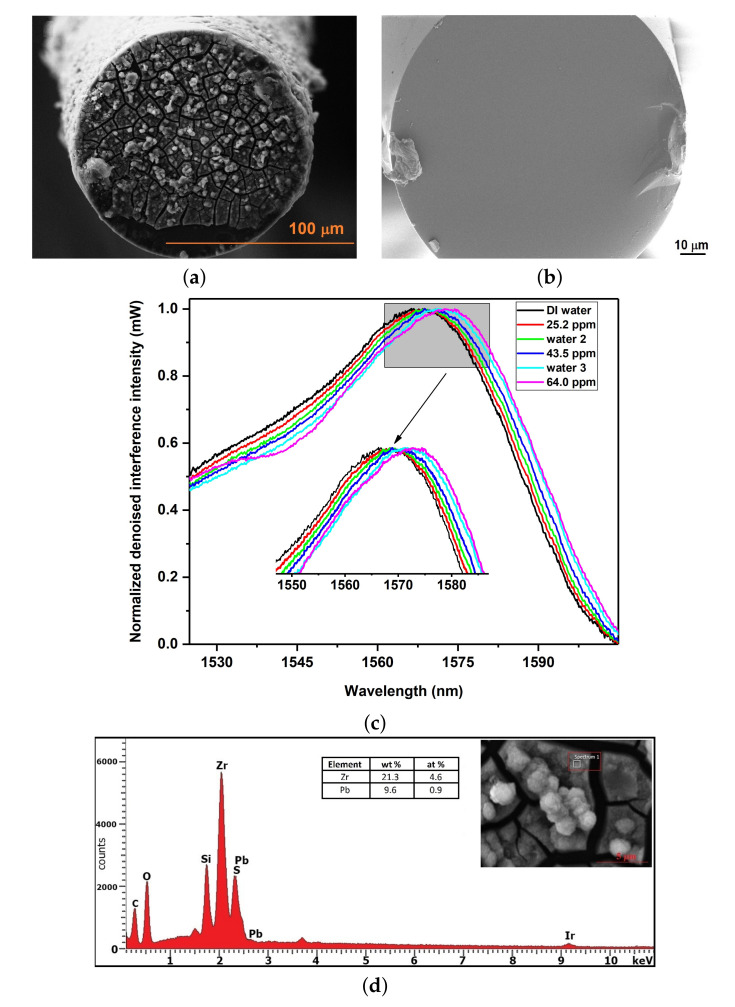
SEM images of (**a**) SO${}_{3}$H-UiO-66(Zr) optical fiber sensing element, and (**b**) bare optical fiber, (**c**) Interferograms in correlation with a lead concentration in DI water, and (**d**) EDX spectra of SO${}_{3}$H-UiO-66(Zr) optical fiber sensing element after the lead uptake.

After successfully validating the impregnation of lead within SO${}_{3}$H-UiO-66(Zr), the coated element at the tip of optical fiber was utilized as a chemical sensor for the rapid (a few milliseconds) detection of lead in DI water. Figure 3a illustrates the SEM image of a deposited sensing element of sulfonated SO${}_{3}$H-UiO-66(Zr) at the tip of optical fiber adjacent to a bare optical fiber in Figure 3b. The interference of two reflected beams was recorded using an optical spectrum analyzer (OSA, Ando Japan, AQ6317B, 600–1750 nm). An Agilent 83438A Erbium ASE (Agilent Technologies, Santa Rosa, CA, USA) was used as the light source with the wavelength range of 1500–1600 nm. The OSA was set on a continuous scan mode to record the interference signals every 5 s with a high sensitivity and 0.1 nm resolution.

The average of all signals (100 trials) for each concentration was used as the final interference signal for a specific concentration. The fast response of the sensing element towards lead uptake was withdrawn by comparing the shape of signals at different sweeps. Figure 3c shows the spectral positions of the interferogram peak (normalized denoised interference intensity vs. wavelength) generated by SO${}_{3}$H-UiO-66(Zr) sensing element at different lead concentrations. Briefly, the sensor was first placed in DI water for 5 min to observe the pattern of sensing element upon the exposure to DI water. The same procedure was repeated after immersing the sensor in different lead solutions (“water 2” and “water 3” with 25.2 ppm and 43.5 ppm lead contents, respectively). Upon the introduction of lead nitrate solutions, the position of interferogram peak shifted to longer wavelengths. This indicated an increase in the lead adsorption by SO${}_{3}$H-UiO-66(Zr) sensing element and, thus, the optical thickness.

Energy-Dispersive X-ray (EDX) analysis was performed on the optical fiber sensing element after the lead uptake process, in order to confirm the attachment of lead with SO${}_{3}$H-UiO-66(Zr). As shown in Figure 3d, despite some detection difficulty due to the similarity of the energy of S-Kα and Pb-Mα (2.309 vs. 2.342 keV, respectively), zirconium (Zr) (as the main constituent of Zr-based MOF SO${}_{3}$H-UiO-66) had 21.3% weight while Pb possessed 9.6% level in the samples with higher ppm. The presence of sulfur (S) confirmed the SO${}_{3}$H-UiO-66(Zr) structure.

As the reference for lead level in drinking water is 5 µg/L (5 ppb) [42], precision evaluation studies should be conducted to further overcome the detection limit. Nevertheless, optical fibers and SO${}_{3}$H-UiO-66(Zr) are stable within the range of pH 2–9 [43,44]. Yet, the remaining challenge is to consider the sensor in very harsh environments for prolonged periods of time where the wastewater might be extremely acidic or basic. Although the optical fiber sensors were not designed explicitly for this purpose, there is an opportunity to trigger the release of the guest species (Pb${}^{2+}$) using light at a specific wavelength [45]. The pH change [46], ligand exchange [47], and ethanol regeneration solvent [48] have been proposed to recycle MOFs for further usages.

### 3.4. Mechanism of Pb${}^{2+}$ Adsorption

**Figure 4:**
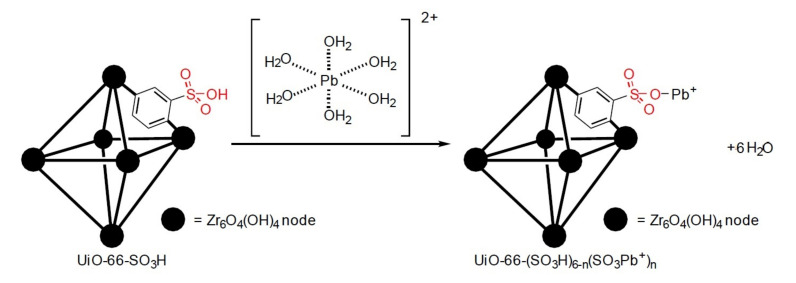
Diagram of adsorption of [Pb(OH${}_{2}$)${}_{6}$]${}^{2+}$ on SO${}_{3}$H-UiO-66(Zr) in water

Figure 4 shows the chemistry mechanism where the sensing material coordinates with Pb${}^{2+}$ ions via *n* sulfonate groups, *n*≤ 6.

As per our data, Pb adsorption occurrs in a S-O-Pb${}^{+}$ bonding manner. There are 10 lone pairs of electrons present in the oxygens of sulfonic acid group, where the tetrahedral R-SO${}_{3}$ geometry would prohibit the direct S-Pb bonding. In fact, the presence of a crystalline phase identical to Pb(SO${}_{4}$) is due to the coordination of Pb to SO${}_{3}^{-}$ group with the *O*-carbon of 2-sulfonoterepthalic acid replacing one *O* in Pb(SO${}_{4}$). The rapid uptake of Pb, shown from both the batch adsorption measurements and the optical fiber results, leads to both weak coordination and strong electrostatic attraction between Pb and R-O${}^{-}$. The speciation of Pb(NO${}_{3}$)${}_{2}$ in aqueous solutions at the native pH of ∼5 is mainly Pb${}^{2+}$, that exists within a solvation shell of approximately 6 H${}_{2}$O molecules. The adsorptions would, therefore, have the following predominant reactions [49]:

(3) $$R-SO_{2}OH_{\left(s\right)}+Pb_{\left(aq\right)}^{2+}⟶R-SO_{3}^{-}+Pb_{\left(aq\right)}^{2+}+H_{3}O_{\left(aq\right)}^{+}⟶R-SO_{2}O-Pb_{s}^{+}$$

(4) $$R-SO_{2}OH_{\left(s\right)}+Pb_{\left(aq\right)}^{2+}⟶R-SO_{3}^{-}+Pb_{\left(aq\right)}^{2+}+H_{3}O_{\left(aq\right)}^{+}⟶R-SO_{3}^{-}...Pb_{s}^{2+}$$

Equation (3) demonstrates an electrostatic attraction to a singular sulfonate *O*, while Equation (4) presents the coordination between the delocalized sulfonate e${}^{-}$ and Pb${}^{2+}$. These are shown as a three-step process. The immersion of the MOF into water dissociates the sulfonic acid H${}^{+}$ rapidly, after which lead is attracted to the sulfonate groups of SO${}_{3}$H-UiO-66(Zr). Due to the rapid uptake, it is not expected that Pb(O.H.)${}^{-}$ is adsorbed onto the sulfonates, as its fractional abundance at pH 5–6 is low relative to the adsorption phenomenon [50].

## 4. Conclusions

The rapid detection of Pb${}^{2+}$ by MOF-coated optical fibers was introduced for the first time. A solvothermal synthesized sulfonic acid functionalized MOF, SO${}_{3}$H-UiO-66(Zr), demonstrated the rapid uptake of lead from Pb(NO${}_{3}$)${}_{2}$ solution. The analysis of ICP-OES intensities showed the uptake capacity of SO${}_{3}$H-UiO-66(Zr) as 32.77 mg/g (R${}^{2}$ = 0.997). This MOF was grown on an OH-functionalized SMF-28 conventional single-mode optical fiber that acted as a detector for Pb${}^{2+}$ at the ppm-level concentrations. Spectral interferograms indicated the detection of Pb${}^{2+}$ down to 25.2 ppm. Such a MOF optical fiber can be implemented as a device for simple/abrupt on-site detection of aqueous Pb${}^{2+}$ or possibly other ions at ppm or sub-ppm levels. We envisage that this type of sensor would offer a novel and more effective composite to harvest heavy metals from contaminated water for clean water supply.

Further studies are currently undergoing to investigate the interference effects of a matrix containing more than one cation and anion in water, as other transition metal (II) ions may give a ’false’ Pb${}^{2+}$ detection reading.

## Figures and Tables

**Table 1:** Langmuir and Freundlich isotherm constants for the uptake of lead (II) cation at room temperature.

Langmuir Constants			Freundlich Constants		
$q_{m}$ (mg/g)	$K_{L}$ (mg/L)	R ${}^{2}$	$K_{f}$	* n *	*R* ${}^{2}$
32.77	6.07	0.95	5.51	1.66	0.99

## Data Availability

The data presented in this study are available on request from the corresponding author.

## Appendix A

### Appendix A.1. Sample Preparation and Analysis Conditions for X-ray Diffraction (XRD) Characterization

The sample was dry ground in a boron carbide mortar and pestle before being loaded into a low volume Si zero background sample holder. A “Bruker D8 Advance A25” X-ray Diffractometer operating under CuKα radiation (40 kV, 40 mA) and equipped with a Lynx Eye XE-T detector was employed to obtain the XRD patterns. The sample was scanned over the 2θ range of 5° to 85° at a step size of 0.02° and a count time of 1.6 s per step and spun at 15 RPM during the data collection.

Analyses were performed on the collected XRD data using the Bruker XRD search match program EVA™ v5. Crystalline phases were identified using the ICDD-JCPDS powder diffraction database. Pawley analyses were performed on the data using the Bruker TOPAS™ v6 program to determine lattice parameters and crystallite size (Table A1). Background signal was described using a combination of Chebyshev polynomial linear interpolation function and 1/*x* function. Cell parameters, vertical sample displacement, full peak width at half maximum, and peak scale factor were all refined. Error ranges were calculated based on three estimated standard deviations as calculated by TOPAS. A diffractogram pattern of SO${}_{3}$Pb-UiO-66(Zr) at 2θ region (5–65°) superimposed over the simulated diffractograms of UiO-66 and PbSO${}_{4}$ anglesite is shown in Figure A1.

**Table A1:** Phase summary of SO${}_{3}$H-UiO-66(Zr).

	Lattice Parameter	Crystallite Size	Phase
**24 h**	NP (amorphous)	NP (amorphous)	UiO-66, except the (0 2 2) peak at ∼12° 2θ
**48 h**	20.678 ± 0.001 Å	170 ± 11 nm	UiO-66, except the (0 2 2) peak at ∼12° 2θ

### Appendix A.2. N 2 Gas Porosimetry

Gas adsorption isotherms were measured for pressure within the range 0–120 kPa by a volumetric approach using a Micrometrics ASAP 2420 instrument. All samples were transferred to pre-dried and weighted analysis tubes and sealed with Transcal stoppers. They were evacuated and activated under a dynamic vacuum at 10${}^{-6}$ Torr at 140 °C for 8 h. Ultra-high purity N${}_{2}$ was used for the experiments. N${}_{2}$ adsorption and desorption measurements were conducted at 77 K. Surface area measurements were performed on N${}_{2}$ isotherms at 77 K using the Brunauer-Emmer-Teller (BET) model with increasing adsorption values of 0.005 to 0.2.

### Appendix A.3. Inductively Coupled Plasma-Optical Emission Spectrometry (Icp-Oes) Analysis Method

The samples were analyzed on an as-received basis by acidifying to 5(wt)% HNO${}_{3}$. The solutions were then analyzed by using Varian 730-ES axial ICP-OES. Certified multi-element solutions were used to check the accuracy of the calibration standards and the method.

### Appendix A.5. Optical Methodology

The interference signal was processed in MATLAB [39] using a special two-stage signal processing algorithm. The first part of the algorithm used Daubechies Wave Transform (DWT) to remove noise from the spectrum. Then, the second part of the algorithm performed frequency domain analysis (fast Fourier transform (FFT)) to determine the response of the SO${}_{3}$H-UiO-66(Zr) to lead concentration in DI water. Table A2 shows the raw data (Normalized denoised interference intensity (NDII) vs. Wavelength) obtained form one sensor after signal processing.

**Table A2:** Normalized denoised interference intensity (NDII).

Wavelength	NDII	NDII	NDII	NDII	NDII	NDII
**nm**	**mW**	**mW**	**mW**	**mW**	**mW**	**mW**
	**DI Water**	**25.2 ppm**	**Water 2**	**43.5 ppm**	**Water 3**	**64.0 ppm**
1524.9	0.51753	0.49844	0.47935	0.47701	0.45939	0.49888
1525.02	0.50941	0.49544	0.48147	0.47317	0.46254	0.49268
1525.14	0.50486	0.49111	0.47736	0.47044	0.45853	0.49062
1525.26	0.51375	0.49504	0.47633	0.47243	0.46196	0.49004
1525.38	0.51785	0.49733	0.47681	0.47322	0.46254	0.49151
1525.5	0.52234	0.50184	0.48134	0.47885	0.4703	0.49534
1525.619	0.52747	0.50358	0.47969	0.47626	0.46483	0.49888
1525.739	0.52365	0.50136	0.47907	0.47243	0.46512	0.49652
1525.859	0.51843	0.50033	0.48223	0.47731	0.4654	0.49681
1525.979	0.52216	0.50271	0.48326	0.47507	0.46655	0.49859
1526.099	0.51376	0.5012	0.48864	0.48326	0.46943	0.49947
1526.219	0.5233	0.50652	0.48974	0.4814	0.4703	0.50036
1526.339	0.52529	0.50493	0.48457	0.4814	0.46799	0.49918
1526.458	0.52852	0.50986	0.4912	0.48256	0.47001	0.50273
1526.578	0.52389	0.50668	0.48947	0.47955	0.46828	0.50066
1526.698	0.5253	0.50946	0.49362	0.48682	0.46943	0.50333
1526.818	0.52945	0.51289	0.49633	0.48466	0.47347	0.50749
1526.938	0.52949	0.51225	0.49501	0.48581	0.47376	0.506
1527.058	0.53234	0.51329	0.49424	0.48667	0.4755	0.50541
1527.178	0.53325	0.51465	0.49605	0.48657	0.4755	0.506
1527.298	0.53873	0.51937	0.50001	0.49241	0.47898	0.51137
1527.418	0.54171	0.52194	0.50217	0.49306	0.48218	0.51316
1527.537	0.53698	0.51937	0.50176	0.49316	0.48276	0.51047
1527.657	0.54221	0.52226	0.50231	0.49463	0.48247	0.51257
1527.777	0.5467	0.52468	0.50266	0.49412	0.48218	0.51346
1527.897	0.54672	0.52637	0.50602	0.49838	0.48334	0.51436
1528.017	0.54467	0.52685	0.50903	0.49883	0.48684	0.51766
1528.137	0.54123	0.52387	0.50651	0.49767	0.48363	0.51406
1528.257	0.5441	0.52492	0.50574	0.49726	0.48188	0.51496
1528.377	0.54442	0.52669	0.50896	0.49741	0.48392	0.51466
1528.496	0.54698	0.53057	0.51416	0.50437	0.48684	0.51856
1528.616	0.54976	0.53178	0.5138	0.50335	0.48889	0.52006
1528.736	0.55104	0.53137	0.5117	0.5031	0.48684	0.51856
1528.856	0.54944	0.53113	0.51282	0.50452	0.48772	0.52036
1528.976	0.55047	0.53235	0.51423	0.503	0.48801	0.52006
1529.216	0.55852	0.53884	0.51916	0.50962	0.49475	0.527
1529.336	0.555	0.53648	0.51796	0.50804	0.49182	0.52338
1529.456	0.5608	0.5403	0.5198	0.51024	0.49387	0.52579
1529.575	0.5663	0.54471	0.52312	0.51234	0.49681	0.52942
1529.695	0.56668	0.54405	0.52142	0.51275	0.49505	0.5273
1529.815	0.56786	0.5456	0.52334	0.51357	0.49888	0.53003
1529.935	0.56246	0.54226	0.52206	0.51285	0.49593	0.52821
1530.055	0.55992	0.54177	0.52362	0.51111	0.49593	0.5264
1530.175	0.56607	0.54683	0.52759	0.51613	0.50094	0.52851
1530.295	0.56429	0.5474	0.53051	0.51886	0.50153	0.53063
1530.415	0.56896	0.5497	0.53044	0.51819	0.5036	0.53154
1530.534	0.56563	0.54757	0.52951	0.51875	0.50094	0.53033
1529.096	0.55331	0.53405	0.51479	0.50575	0.49123	0.52277
1530.654	0.56891	0.54978	0.53065	0.51839	0.50272	0.53185
1530.774	0.5686	0.55084	0.53308	0.52056	0.5042	0.53215
1530.894	0.57401	0.55429	0.53457	0.52432	0.50538	0.53549
1531.014	0.57661	0.5547	0.53279	0.52329	0.50509	0.53549
1531.134	0.57067	0.55273	0.53479	0.52247	0.50568	0.53428
1531.254	0.57597	0.5552	0.53443	0.52221	0.50686	0.5361
1531.374	0.58032	0.55866	0.537	0.52443	0.50746	0.5361
1531.494	0.57778	0.5575	0.53722	0.52593	0.50746	0.53702
1531.613	0.58055	0.56064	0.54073	0.52784	0.51073	0.53885
1531.733	0.57708	0.55808	0.53908	0.52582	0.50835	0.53732
1531.853	0.58131	0.56113	0.54095	0.5265	0.51043	0.53732
1531.973	0.57906	0.56072	0.54238	0.52909	0.51132	0.53915
1532.093	0.58306	0.56477	0.54648	0.53356	0.51431	0.54129
1532.213	0.58573	0.56809	0.55045	0.53616	0.51849	0.54435
1532.333	0.58018	0.56477	0.54936	0.53512	0.51699	0.54129
1532.453	0.58271	0.56535	0.54799	0.53397	0.5164	0.54098
1532.572	0.58962	0.57	0.55038	0.53679	0.51969	0.54496
1532.692	0.5882	0.57175	0.5553	0.54196	0.52239	0.54619
1532.812	0.59301	0.57575	0.55849	0.54468	0.5257	0.55141
1532.932	0.58986	0.572	0.55414	0.54076	0.52089	0.5465
1533.052	0.5902	0.57141	0.55262	0.53929	0.52239	0.54588
1533.172	0.59337	0.57575	0.55813	0.54311	0.5257	0.5468
1533.292	0.59498	0.57608	0.55718	0.54474	0.52449	0.5468
1533.412	0.59503	0.57658	0.55813	0.54364	0.5263	0.54926
1533.531	0.5945	0.57566	0.55682	0.54238	0.52359	0.54742
1533.651	0.59242	0.57433	0.55624	0.54269	0.52299	0.54496
1533.771	0.59648	0.57901	0.56154	0.54736	0.52901	0.54711
1533.891	0.59699	0.57934	0.56169	0.5482	0.52871	0.54803
1534.011	0.59912	0.58303	0.56694	0.55142	0.53204	0.55111
1534.131	0.60068	0.58286	0.56504	0.55179	0.53204	0.54895
1534.251	0.59938	0.58261	0.56584	0.553	0.53355	0.5508
1534.371	0.60338	0.58622	0.56906	0.55628	0.53598	0.55234
1534.49	0.604	0.58697	0.56994	0.55553	0.53598	0.55234
1534.61	0.60676	0.58967	0.57258	0.55808	0.53962	0.5545
1534.73	0.60637	0.5879	0.56943	0.55866	0.53689	0.55326
1534.85	0.60773	0.58748	0.56723	0.55601	0.53719	0.55203
1534.97	0.60591	0.58807	0.57023	0.55749	0.53719	0.55234
1535.09	0.60928	0.59034	0.5714	0.55739	0.53841	0.55265
1535.21	0.61246	0.59296	0.57346	0.55998	0.54145	0.55419
1535.33	0.61354	0.59119	0.56884	0.55845	0.53719	0.55111
1535.45	0.61025	0.58874	0.56723	0.55575	0.53689	0.54772
1535.569	0.61255	0.59161	0.57067	0.55792	0.53932	0.54895
1535.689	0.61792	0.59668	0.57544	0.56376	0.54328	0.55357
1535.809	0.61768	0.59829	0.5789	0.56647	0.54756	0.5579
1535.929	0.61427	0.59541	0.57655	0.56429	0.5442	0.55296
1536.049	0.61592	0.59693	0.57794	0.56386	0.54603	0.55357
1536.169	0.62022	0.60015	0.58008	0.5693	0.54786	0.55419
1536.289	0.61962	0.60177	0.58392	0.57256	0.55247	0.5579
1536.409	0.61945	0.60202	0.58459	0.57267	0.55339	0.55914
1536.528	0.61952	0.59888	0.57824	0.56903	0.54878	0.55512
1536.648	0.61907	0.60024	0.58141	0.57058	0.5497	0.55512
1536.768	0.6239	0.60321	0.58252	0.57192	0.55124	0.55481
1536.888	0.62285	0.60372	0.58459	0.5708	0.55124	0.55419
1537.008	0.62361	0.60432	0.58503	0.57176	0.5537	0.55728
1537.128	0.62407	0.60355	0.58303	0.5701	0.55154	0.55419
1537.248	0.62814	0.60662	0.5851	0.57085	0.55216	0.55419
1537.368	0.62787	0.60704	0.58621	0.5754	0.55308	0.55542
1537.488	0.63204	0.60935	0.58666	0.57503	0.55647	0.55573
1537.607	0.63287	0.61106	0.58925	0.5804	0.55924	0.55883
1537.727	0.63163	0.61063	0.58963	0.58013	0.55832	0.55666
1537.847	0.63184	0.61088	0.58992	0.58078	0.55893	0.55635
1537.967	0.63351	0.61242	0.59133	0.58169	0.55955	0.5579
1538.087	0.63312	0.61491	0.5967	0.58282	0.56264	0.55914
1538.207	0.63598	0.61757	0.59916	0.58817	0.56481	0.56038
1538.327	0.63336	0.61697	0.60058	0.58477	0.56295	0.56255
1538.447	0.63753	0.61868	0.59983	0.58747	0.56481	0.56255
1538.566	0.63928	0.61765	0.59602	0.58758	0.56512	0.55914
1538.686	0.64145	0.62083	0.60021	0.59072	0.56792	0.56038
1538.806	0.64252	0.62402	0.60552	0.59305	0.56947	0.56348
1538.926	0.63883	0.61963	0.60043	0.58823	0.56699	0.56069
1539.046	0.64447	0.62144	0.59841	0.58925	0.56761	0.55976
1539.166	0.63852	0.62015	0.60178	0.58925	0.56885	0.55945
1539.286	0.64516	0.62583	0.6065	0.59273	0.5729	0.561
1539.406	0.6517	0.62929	0.60688	0.59735	0.5729	0.56193
1539.526	0.65148	0.62903	0.60658	0.59506	0.57509	0.56131
1539.645	0.64663	0.62773	0.60883	0.59588	0.57352	0.55976
1539.765	0.64923	0.62903	0.60883	0.59833	0.57477	0.56224
1539.885	0.65419	0.63328	0.61237	0.60139	0.57696	0.56441
1540.005	0.65544	0.63553	0.61562	0.60434	0.57947	0.56784
1540.125	0.65478	0.63414	0.6135	0.60084	0.5779	0.56659
1540.245	0.65234	0.63198	0.61162	0.60193	0.57446	0.56379
1540.365	0.65543	0.63371	0.61199	0.60139	0.57821	0.56317
1540.484	0.65678	0.63771	0.61864	0.60434	0.57978	0.56628
1540.604	0.65643	0.6364	0.61637	0.60544	0.58261	0.56846
1540.724	0.66255	0.63814	0.61373	0.60352	0.57978	0.56472
1540.844	0.65757	0.63701	0.61645	0.60478	0.58072	0.56628
1540.964	0.6608	0.63919	0.61758	0.60533	0.58418	0.56753
1541.084	0.66163	0.64093	0.62023	0.60642	0.58607	0.56784
1541.204	0.66687	0.64416	0.62145	0.60878	0.58985	0.56784
1541.324	0.66888	0.64714	0.6254	0.61049	0.59017	0.57033
1541.444	0.6667	0.64635	0.626	0.61401	0.59207	0.5719
1541.563	0.66733	0.6453	0.62327	0.61137	0.58922	0.57127
1541.683	0.6656	0.64504	0.62448	0.61208	0.59143	0.5719
1541.803	0.67077	0.64968	0.62859	0.61467	0.59397	0.57252
1541.923	0.67036	0.64845	0.62654	0.61462	0.59428	0.57471
1542.043	0.67384	0.65152	0.6292	0.6171	0.59492	0.57534
1542.163	0.6695	0.64924	0.62898	0.6155	0.59555	0.57503
1542.283	0.66931	0.65082	0.63233	0.61809	0.59428	0.57377
1542.403	0.67271	0.65275	0.63279	0.61688	0.59618	0.57534
1542.522	0.68003	0.65645	0.63287	0.62009	0.59936	0.57691
1542.642	0.68196	0.65768	0.6334	0.62347	0.60159	0.57754
1542.762	0.67908	0.65609	0.6331	0.61809	0.60127	0.57817
1542.882	0.67796	0.65733	0.6367	0.62286	0.60478	0.57879
1543.002	0.68338	0.66165	0.63992	0.62485	0.6051	0.58037
1543.122	0.6828	0.66263	0.64246	0.62674	0.60702	0.58194
1543.242	0.69194	0.66785	0.64376	0.6307	0.6099	0.58383
1543.362	0.68645	0.66484	0.64323	0.62814	0.60894	0.58509
1543.482	0.68584	0.66519	0.64454	0.63181	0.6099	0.58636
1543.601	0.69129	0.6683	0.64531	0.63209	0.61214	0.58794
1543.721	0.69079	0.66847	0.64615	0.63394	0.61086	0.58762
1543.841	0.6961	0.67248	0.64886	0.63617	0.61632	0.59238
1543.961	0.69443	0.67141	0.64839	0.63545	0.61407	0.59016
1544.081	0.6941	0.67256	0.65102	0.6364	0.61471	0.59269
1544.201	0.69518	0.67337	0.65156	0.63645	0.616	0.59142
1544.321	0.69867	0.67783	0.65699	0.64178	0.6189	0.59651
1544.441	0.70701	0.68266	0.65831	0.64296	0.62407	0.59874
1544.56	0.70144	0.67836	0.65528	0.64257	0.62245	0.5981
1544.68	0.70117	0.67881	0.65645	0.64313	0.62213	0.59683
1544.8	0.70591	0.6832	0.66049	0.646	0.62569	0.5997
1544.92	0.70794	0.68562	0.6633	0.65182	0.62991	0.60385
1545.04	0.70924	0.68732	0.6654	0.65103	0.63024	0.60706
1545.16	0.70735	0.68661	0.66587	0.65091	0.62991	0.60673
1545.28	0.71117	0.68993	0.66869	0.65391	0.63056	0.6093
1545.4	0.71107	0.69074	0.67041	0.65482	0.63284	0.61123
1545.52	0.71115	0.69192	0.67269	0.65862	0.63317	0.61252
1545.639	0.71684	0.6948	0.67276	0.65879	0.6348	0.61413
1545.759	0.71638	0.69426	0.67214	0.658	0.63317	0.61252
1545.879	0.71627	0.69444	0.67261	0.65788	0.63414	0.61316
1545.999	0.71613	0.69598	0.67583	0.65976	0.63643	0.61478
1546.119	0.71838	0.69742	0.67646	0.66084	0.63741	0.6151
1546.239	0.72598	0.70177	0.67756	0.66409	0.64068	0.61898
1546.359	0.72277	0.70123	0.67969	0.66329	0.64166	0.61962
1546.479	0.7235	0.70195	0.6804	0.66569	0.64396	0.62092
1546.598	0.725	0.70404	0.68308	0.66723	0.64659	0.62222
1546.718	0.72222	0.70277	0.68332	0.6686	0.64462	0.62124
1546.838	0.73196	0.7095	0.68704	0.67038	0.64955	0.62709
1546.958	0.72775	0.70668	0.68561	0.67043	0.64922	0.62774
1547.078	0.73353	0.71151	0.68949	0.67313	0.65318	0.63067
1547.198	0.73033	0.71023	0.69013	0.67336	0.65186	0.631
1547.318	0.73222	0.71169	0.69116	0.67502	0.65252	0.63035
1547.438	0.73384	0.71489	0.69594	0.67761	0.65649	0.63525
1547.557	0.7323	0.71352	0.69474	0.67876	0.65285	0.63394
1547.677	0.73636	0.71599	0.69562	0.67836	0.65517	0.63557
1547.797	0.7328	0.71397	0.69514	0.67761	0.65616	0.63361
1547.917	0.73969	0.71901	0.69833	0.68078	0.65948	0.63885
1548.037	0.7416	0.72112	0.70064	0.68436	0.66147	0.64115
1548.157	0.74294	0.72287	0.7028	0.68587	0.66413	0.64181
1548.277	0.74673	0.72645	0.70617	0.68824	0.66646	0.64609
1548.397	0.7439	0.72323	0.70256	0.68592	0.66413	0.64279
1548.516	0.74703	0.72728	0.70753	0.69033	0.66913	0.64708
1548.636	0.75317	0.73236	0.71155	0.69469	0.67449	0.65369
1548.756	0.75597	0.73505	0.71413	0.69795	0.67449	0.65668
1548.876	0.75013	0.73338	0.71663	0.69755	0.67583	0.65768
1548.996	0.75196	0.73236	0.71276	0.69906	0.67248	0.65668
1549.116	0.7561	0.73681	0.71752	0.70064	0.67785	0.66134
1549.236	0.75338	0.73662	0.71986	0.70251	0.67818	0.66067
1549.356	0.75768	0.73885	0.72002	0.70316	0.67886	0.66334
1549.475	0.75861	0.74191	0.72521	0.70767	0.68256	0.66735
1549.595	0.76076	0.74043	0.7201	0.70538	0.67987	0.66468
1549.715	0.75874	0.74108	0.72342	0.70673	0.68256	0.66568
1549.835	0.76532	0.74685	0.72838	0.71249	0.68459	0.6697
1549.955	0.77077	0.75059	0.73041	0.71426	0.69102	0.67642
1550.075	0.76692	0.74891	0.7309	0.71503	0.69102	0.67608
1550.195	0.76662	0.74807	0.72952	0.71467	0.69102	0.67743
1550.315	0.76931	0.75227	0.73523	0.71733	0.69611	0.68114
1550.435	0.77103	0.75415	0.73727	0.72206	0.69782	0.68317
1550.554	0.77644	0.75997	0.7435	0.72521	0.70294	0.69029
1550.674	0.77855	0.76053	0.74251	0.72604	0.70157	0.69199
1550.794	0.77572	0.75846	0.7412	0.72467	0.70089	0.69165
1550.914	0.77438	0.75865	0.74292	0.72723	0.70259	0.69369
1551.034	0.77538	0.761	0.74662	0.72931	0.70328	0.69574
1551.154	0.78577	0.76751	0.74925	0.73295	0.70979	0.70189
1551.274	0.77862	0.76373	0.74884	0.73325	0.70704	0.69881
1551.394	0.78722	0.76647	0.74572	0.73211	0.70807	0.70087
1551.513	0.78558	0.76779	0.75	0.73606	0.70842	0.70327
1551.633	0.78389	0.76789	0.75189	0.73719	0.70979	0.70395
1551.753	0.79135	0.77319	0.75503	0.73983	0.71461	0.70911
1551.873	0.79585	0.77594	0.75603	0.74379	0.71771	0.71117
1551.993	0.79432	0.77509	0.75586	0.74283	0.71806	0.71428
1552.113	0.7939	0.77575	0.7576	0.74481	0.71909	0.71394
1552.233	0.7995	0.77946	0.75942	0.74547	0.72186	0.71878
1552.353	0.80633	0.78795	0.76957	0.75489	0.73054	0.72573
1552.473	0.80423	0.78661	0.76899	0.75543	0.73228	0.72712
1552.592	0.80431	0.78853	0.77275	0.75513	0.73124	0.72921
1552.712	0.80448	0.78757	0.77066	0.75604	0.72915	0.73096
1552.832	0.80695	0.78968	0.77241	0.75956	0.73368	0.73586
1552.952	0.81271	0.79591	0.77911	0.76472	0.73752	0.73831
1553.072	0.81535	0.79639	0.77743	0.76442	0.73787	0.74042
1553.192	0.81192	0.79514	0.77836	0.76369	0.73647	0.73866
1553.312	0.81002	0.79553	0.78104	0.76497	0.73822	0.73936
1553.432	0.80936	0.79659	0.78382	0.76521	0.73998	0.74147
1553.551	0.81399	0.80063	0.78727	0.77119	0.74348	0.74676
1553.671	0.82159	0.80536	0.78913	0.7734	0.7477	0.75029
1553.791	0.82201	0.80671	0.79141	0.77468	0.74947	0.75135
1553.911	0.82297	0.80584	0.78871	0.77309	0.75088	0.75348
1554.031	0.82577	0.80952	0.79327	0.77812	0.75477	0.75632
1554.151	0.82942	0.81457	0.79972	0.78353	0.75866	0.76023
1554.271	0.83184	0.81612	0.8004	0.78409	0.75902	0.76487
1554.391	0.83197	0.81661	0.80125	0.78563	0.75866	0.76701
1554.51	0.82876	0.81428	0.7998	0.78403	0.75866	0.76665
1554.63	0.83265	0.81895	0.80525	0.78754	0.76186	0.76916
1554.75	0.83875	0.82217	0.80559	0.7886	0.76328	0.76987
1554.87	0.84071	0.8252	0.80969	0.79256	0.76471	0.7767
1554.99	0.83796	0.82344	0.80892	0.79429	0.76649	0.77562
1555.11	0.83917	0.8249	0.81063	0.79212	0.76684	0.77598
1555.23	0.84242	0.82794	0.81346	0.79796	0.76863	0.77886
1555.35	0.8488	0.83216	0.81552	0.79995	0.77184	0.78138
1555.469	0.84929	0.83481	0.82033	0.8045	0.77579	0.78536
1555.589	0.85237	0.83609	0.81981	0.80494	0.7783	0.78753
1555.709	0.85114	0.83668	0.82222	0.80644	0.78046	0.78862
1555.829	0.85743	0.84112	0.82481	0.81082	0.78334	0.79188
1555.949	0.86059	0.84369	0.82679	0.80982	0.78514	0.79479
1556.069	0.85977	0.84497	0.83017	0.81496	0.78658	0.79698
1556.189	0.85971	0.84507	0.83043	0.81427	0.78948	0.7999
1556.309	0.86231	0.84784	0.83337	0.81659	0.78984	0.80173
1556.429	0.86566	0.84943	0.8332	0.81968	0.78984	0.80173
1556.548	0.86356	0.85003	0.8365	0.8193	0.79092	0.80429
1556.668	0.86858	0.8538	0.83902	0.8229	0.7931	0.80795
1556.788	0.87084	0.8551	0.83936	0.82302	0.79673	0.80795
1556.908	0.87313	0.85838	0.84363	0.82758	0.79964	0.81126
1557.028	0.87394	0.85918	0.84442	0.82929	0.8	0.81163
1557.148	0.87538	0.86108	0.84678	0.82998	0.8011	0.81568
1557.268	0.88238	0.86668	0.85098	0.83373	0.80438	0.81863
1557.388	0.88802	0.87099	0.85396	0.83863	0.80914	0.82159
1557.507	0.89148	0.8725	0.85352	0.8415	0.81134	0.82345
1557.627	0.88677	0.87019	0.85361	0.83787	0.81134	0.82345
1557.747	0.89029	0.873	0.85571	0.84182	0.81317	0.82419
1557.867	0.89629	0.87895	0.86161	0.84412	0.81795	0.8279
1557.987	0.89623	0.87976	0.86329	0.84489	0.82164	0.83013
1558.107	0.89502	0.88238	0.86974	0.85156	0.82423	0.83684
1558.227	0.89439	0.88127	0.86815	0.84969	0.82312	0.83684
1558.347	0.90021	0.88471	0.86921	0.85233	0.8246	0.83684
1558.467	0.90288	0.88755	0.87222	0.85246	0.82682	0.83984
1558.586	0.90357	0.88927	0.87497	0.85877	0.82979	0.84246
1558.706	0.91208	0.89344	0.8748	0.85677	0.8309	0.84246
1558.826	0.90884	0.89364	0.87844	0.86245	0.83462	0.84509
1558.946	0.91572	0.89904	0.88236	0.86484	0.83909	0.8496
1559.066	0.92115	0.9018	0.88245	0.86504	0.83947	0.8496
1559.186	0.92187	0.90426	0.88665	0.87029	0.84021	0.85413
1559.306	0.92322	0.90641	0.8896	0.8725	0.8462	0.85602
1559.426	0.92339	0.90753	0.89167	0.87699	0.8492	0.85753
1559.545	0.92797	0.91031	0.89265	0.87915	0.85409	0.8636
1559.665	0.92835	0.91041	0.89247	0.87908	0.85259	0.86246
1559.785	0.93072	0.9138	0.89688	0.8815	0.85711	0.8655
1559.905	0.93318	0.9171	0.90102	0.88333	0.85975	0.8693
1560.025	0.93376	0.91906	0.90436	0.88602	0.85975	0.86969
1560.145	0.9364	0.92133	0.90626	0.88818	0.86429	0.87274
1560.265	0.93608	0.9204	0.90472	0.8868	0.8624	0.87121
1560.385	0.93648	0.92123	0.90598	0.89022	0.86392	0.87159
1560.505	0.94176	0.9265	0.91124	0.89213	0.86505	0.87503
1560.624	0.94274	0.92785	0.91296	0.89555	0.86847	0.87809
1560.744	0.94275	0.92858	0.91441	0.89773	0.87075	0.88193
1560.864	0.94308	0.92879	0.9145	0.89991	0.87227	0.87809
1560.984	0.94631	0.93159	0.91687	0.90308	0.87266	0.8827
1561.104	0.94526	0.93325	0.92124	0.90302	0.87647	0.88385
1561.224	0.95679	0.93888	0.92097	0.90806	0.88259	0.88847
1561.344	0.95615	0.94066	0.92517	0.90892	0.88489	0.88963
1561.464	0.95451	0.93961	0.92471	0.90925	0.88297	0.89002
1561.583	0.95615	0.94139	0.92663	0.91211	0.88604	0.89388
1561.703	0.95373	0.9416	0.92947	0.91264	0.88835	0.89272
1561.823	0.95509	0.94379	0.93249	0.91591	0.8922	0.89775
1561.943	0.95365	0.94243	0.93121	0.91711	0.8895	0.89775
1562.063	0.95803	0.94421	0.93039	0.91598	0.88874	0.8962
1562.183	0.95876	0.94526	0.93176	0.91564	0.89105	0.89737
1562.303	0.96019	0.94662	0.93305	0.92045	0.89452	0.89853
1562.423	0.96544	0.95145	0.93746	0.92139	0.89683	0.9028
1562.542	0.96202	0.95029	0.93856	0.92279	0.89722	0.90164
1562.662	0.96345	0.95239	0.94133	0.92514	0.90303	0.90436
1562.782	0.96405	0.95292	0.94179	0.92682	0.90303	0.90475
1562.902	0.96694	0.95418	0.94142	0.92803	0.90381	0.9067
1563.022	0.97088	0.95892	0.94696	0.93139	0.90964	0.91177
1563.142	0.97729	0.96125	0.94521	0.93327	0.91042	0.91099
1563.262	0.98311	0.96601	0.94891	0.93698	0.91588	0.91412
1563.382	0.97743	0.96326	0.94909	0.93638	0.91471	0.91334
1563.501	0.98363	0.96738	0.95113	0.93881	0.9198	0.91568
1563.621	0.97304	0.96283	0.95262	0.93969	0.91549	0.91647
1563.741	0.97548	0.96558	0.95568	0.94097	0.91862	0.91725
1563.861	0.97912	0.96791	0.9567	0.93982	0.92176	0.91961
1563.981	0.97801	0.96601	0.95401	0.93901	0.9194	0.91647
1564.101	0.98781	0.97407	0.96033	0.94436	0.92529	0.92
1564.221	0.98063	0.97067	0.96071	0.94619	0.92529	0.92157
1564.341	0.97838	0.97057	0.96276	0.94762	0.92765	0.92393
1564.461	0.98368	0.97322	0.96276	0.94898	0.92883	0.92354
1564.58	0.9874	0.97695	0.9665	0.9534	0.93278	0.92472
1564.7	0.99038	0.98036	0.97034	0.9577	0.93831	0.93104
1564.82	0.9892	0.97855	0.9679	0.95681	0.93633	0.93064
1564.94	0.98566	0.97823	0.9708	0.95872	0.9399	0.93183
1565.06	0.98168	0.97493	0.96818	0.95879	0.93792	0.92867
1565.18	0.9932	0.98261	0.97202	0.96242	0.94347	0.93301
1565.3	0.99244	0.98303	0.97362	0.9618	0.94506	0.9346
1565.42	0.98877	0.98143	0.97409	0.96214	0.94625	0.93618
1565.539	0.99264	0.98421	0.97578	0.96276	0.94705	0.93658
1565.659	0.99326	0.98325	0.97324	0.96132	0.94785	0.93698
1565.779	0.99524	0.98678	0.97832	0.96694	0.95263	0.94175
1565.899	0.99464	0.98549	0.97634	0.96846	0.95183	0.93896
1566.019	0.99779	0.98796	0.97813	0.96585	0.95383	0.94254
1566.139	0.9967	0.98817	0.97964	0.97052	0.95383	0.94214
1566.259	1.001	0.99107	0.98114	0.97382	0.95663	0.94573
1566.379	0.99199	0.98817	0.98435	0.97465	0.95863	0.94932
1566.499	0.99769	0.99107	0.98445	0.97486	0.96064	0.94772
1566.618	0.99921	0.99386	0.98851	0.97893	0.96506	0.95412
1566.738	1.00015	0.99386	0.98757	0.98135	0.96426	0.95452
1566.858	0.99771	0.99472	0.99173	0.98218	0.96909	0.95653
1566.978	0.99288	0.99117	0.98946	0.98412	0.96949	0.95854
1567.098	0.99197	0.99408	0.99619	0.98641	0.97595	0.96498
1567.218	0.99499	0.99526	0.99553	0.98982	0.97393	0.9674
1567.338	0.99414	0.99289	0.99164	0.98336	0.97474	0.96337
1567.458	0.99635	0.99537	0.99439	0.98697	0.97757	0.96619
1567.577	0.99623	0.99332	0.99041	0.98551	0.97636	0.96538
1567.697	0.99897	0.99644	0.99391	0.98836	0.97879	0.9678
1567.817	0.99721	0.99547	0.99373	0.98829	0.97757	0.96699
1567.937	0.99596	0.99451	0.99306	0.98683	0.97717	0.9678
1568.057	0.99614	0.99569	0.99524	0.99058	0.9792	0.96982
1568.177	0.99668	0.99634	0.996	0.99051	0.98326	0.97225
1568.297	0.99786	0.99817	0.99848	0.99211	0.98285	0.97347
1568.417	1.00069	0.99892	0.99715	0.99267	0.98285	0.97185
1568.536	0.99799	0.99795	0.99791	0.99609	0.98814	0.97671
1568.656	0.99838	0.99881	0.99924	0.99902	0.98896	0.97712
1568.776	1.00095	1	0.99905	0.9979	0.99018	0.97996
1568.896	0.99784	0.99892	1	1	0.99222	0.98199
1569.016	0.99719	0.99774	0.99829	0.99553	0.99304	0.97996
1569.136	0.99194	0.99483	0.99772	0.99567	0.991	0.97793
1569.256	0.99605	0.99655	0.99705	0.99595	0.99263	0.98199
1569.376	0.9924	0.99558	0.99876	0.99483	0.99426	0.98281
1569.495	0.98929	0.99236	0.99543	0.99518	0.99222	0.9824
1569.615	0.9903	0.99225	0.9942	0.99211	0.99304	0.98281
1569.735	0.99004	0.99193	0.99382	0.99616	0.99426	0.98403
1569.855	0.9858	0.98967	0.99354	0.99204	0.99222	0.98159
1569.975	0.98847	0.99214	0.99581	0.99462	0.99549	0.98485
1570.095	0.98734	0.99096	0.99458	0.9926	0.99508	0.98566
1570.215	0.98598	0.99085	0.99572	0.99476	0.9959	0.98852
1570.335	0.98584	0.98817	0.9905	0.99476	0.99304	0.98444
1570.455	0.98741	0.99085	0.99429	0.99581	0.99754	0.98975
1570.574	0.98662	0.98989	0.99316	0.99609	0.99631	0.9877
1570.694	0.98788	0.99085	0.99382	0.9972	0.99713	0.99097
1570.814	0.98669	0.98978	0.99287	0.9986	0.99795	0.99302
1570.934	0.99066	0.99096	0.99126	0.99609	0.99672	0.99261
1571.054	0.98401	0.98806	0.99211	0.99797	0.99959	0.99466
1571.174	0.98135	0.98635	0.99135	0.9963	1	0.99548
1571.294	0.98314	0.98796	0.99278	0.99316	0.99959	0.99589
1571.414	0.98806	0.98914	0.99022	0.99448	0.99918	0.99753
1571.533	0.97941	0.98207	0.98473	0.98933	0.99713	0.99261
1571.653	0.97832	0.98228	0.98624	0.99128	0.99959	0.99425
1571.773	0.97741	0.98154	0.98567	0.99177	0.99959	0.99507
1571.893	0.97334	0.97908	0.98482	0.99093	0.99836	0.99753
1572.013	0.97069	0.97738	0.98407	0.98996	0.99754	0.99548
1572.133	0.97226	0.97642	0.98058	0.98745	0.99713	0.99384
1572.253	0.97158	0.97674	0.9819	0.98933	0.99836	0.99794
1572.373	0.97191	0.97695	0.98199	0.99024	0.99795	0.99753
1572.493	0.97258	0.97738	0.98218	0.99204	0.99918	0.99959
1572.612	0.96465	0.97078	0.97691	0.98523	0.99631	0.99836
1572.732	0.96542	0.97248	0.97954	0.9869	0.99795	1
1572.852	0.96262	0.96887	0.97512	0.98752	0.99754	0.99877
1572.972	0.96064	0.9676	0.97456	0.98489	0.99631	0.99794
1573.092	0.95602	0.96463	0.97324	0.98475	0.99426	0.99877
1573.212	0.95367	0.96177	0.96987	0.98108	0.99059	0.99794
1573.332	0.95202	0.95935	0.96668	0.97783	0.99018	0.9963
1573.452	0.9468	0.9566	0.9664	0.97479	0.98896	0.99589
1573.571	0.94577	0.95534	0.96491	0.97665	0.991	0.9963
1573.691	0.95032	0.95766	0.965	0.9772	0.99345	0.9963
1573.811	0.95038	0.95755	0.96472	0.97624	0.99222	0.99753
1573.931	0.94029	0.95092	0.96155	0.973	0.98733	0.99548
1574.051	0.93684	0.9484	0.95996	0.97017	0.98814	0.99179
1574.171	0.94019	0.9504	0.96061	0.97403	0.98936	0.99589
1574.291	0.93787	0.94966	0.96145	0.97176	0.98896	0.99548
1574.411	0.94057	0.9505	0.96043	0.975	0.99018	1
1574.531	0.93297	0.94442	0.95587	0.96969	0.98855	0.99794
1574.65	0.93265	0.94212	0.95159	0.96639	0.98285	0.99179
1574.77	0.93295	0.94306	0.95317	0.96887	0.9861	0.99753
1574.89	0.92962	0.94107	0.95252	0.96777	0.9861	0.99712
1575.01	0.9244	0.93846	0.95252	0.96708	0.98488	0.9963
1575.13	0.92508	0.93607	0.94706	0.96112	0.98326	0.99425
1575.25	0.91493	0.9291	0.94327	0.95544	0.97555	0.98689
1575.37	0.91653	0.92879	0.94105	0.95565	0.97757	0.98689
1575.49	0.90974	0.92443	0.93912	0.95272	0.97515	0.98444
1575.609	0.9104	0.92278	0.93516	0.95142	0.97717	0.98648
1575.729	0.90584	0.91926	0.93268	0.9466	0.9707	0.98118
1575.849	0.90625	0.91864	0.93103	0.947	0.96828	0.98281
1575.969	0.89911	0.9136	0.92809	0.94355	0.96909	0.98077
1576.089	0.89348	0.90969	0.9259	0.94409	0.96868	0.97833
1576.209	0.89142	0.90866	0.9259	0.93867	0.96868	0.97955
1576.329	0.89593	0.90877	0.92161	0.94131	0.96586	0.97833
1576.449	0.89189	0.90661	0.92133	0.93766	0.96225	0.97996
1576.568	0.88046	0.89853	0.9166	0.93152	0.95743	0.97428
1576.688	0.87887	0.89619	0.91351	0.93307	0.95783	0.97387
1576.808	0.878	0.89344	0.90888	0.92581	0.95343	0.97144
1576.928	0.87722	0.89323	0.90924	0.9293	0.95423	0.97104
1577.048	0.87559	0.89029	0.90499	0.92554	0.95303	0.97144
1577.168	0.86178	0.87996	0.89814	0.91965	0.94625	0.9674
1577.288	0.86426	0.87895	0.89364	0.91431	0.94427	0.96337
1577.408	0.85509	0.87522	0.89535	0.91145	0.94347	0.96055
1577.527	0.85992	0.87804	0.89616	0.91524	0.94546	0.96296
1577.647	0.84969	0.87059	0.89149	0.91052	0.93911	0.95814
1577.767	0.84204	0.86278	0.88352	0.9054	0.9308	0.95292
1577.887	0.8372	0.85938	0.88156	0.90136	0.93041	0.94892
1578.007	0.83583	0.85749	0.87915	0.89832	0.92883	0.94733
1578.127	0.83787	0.85838	0.87889	0.89971	0.92962	0.95052
1578.247	0.83912	0.85589	0.87266	0.89806	0.92608	0.94613
1578.367	0.83033	0.85052	0.87071	0.89305	0.92529	0.94493
1578.487	0.82536	0.84547	0.86558	0.88818	0.91784	0.93936
1578.606	0.82465	0.84388	0.86311	0.88792	0.91784	0.93976
1578.726	0.81845	0.84003	0.86161	0.8849	0.91432	0.93737
1578.846	0.81583	0.83806	0.86029	0.88392	0.91549	0.93737
1578.966	0.81443	0.8356	0.85677	0.87673	0.91198	0.93381
1579.086	0.80873	0.82784	0.84695	0.87146	0.90575	0.92788
1579.206	0.80486	0.82451	0.84416	0.86685	0.9007	0.92315
1579.326	0.80134	0.82266	0.84398	0.86776	0.89915	0.92354
1579.446	0.79802	0.82109	0.84416	0.86523	0.89877	0.92118
1579.565	0.78503	0.8102	0.83537	0.85857	0.89374	0.91608
1579.685	0.78422	0.80633	0.82844	0.85342	0.89027	0.91255
1579.805	0.78272	0.80536	0.828	0.85278	0.8872	0.91099
1579.925	0.77712	0.80092	0.82472	0.84963	0.88413	0.90592
1580.045	0.77461	0.79764	0.82067	0.84604	0.88029	0.90202
1580.165	0.77084	0.79438	0.81792	0.84502	0.87762	0.90241
1580.285	0.76761	0.78968	0.81175	0.83984	0.87647	0.89931
1580.405	0.75576	0.78136	0.80696	0.83297	0.86923	0.89272
1580.525	0.75866	0.78089	0.80312	0.82789	0.86619	0.88847
1580.644	0.75028	0.77632	0.80236	0.82745	0.86429	0.88847
1580.764	0.74705	0.773	0.79895	0.8246	0.8624	0.88539
1580.884	0.74328	0.76921	0.79514	0.82113	0.86051	0.88308
1581.004	0.74383	0.76534	0.78685	0.81615	0.8507	0.87656
1581.124	0.73345	0.75771	0.78197	0.80994	0.8477	0.86702
1581.244	0.72756	0.75405	0.78054	0.80737	0.84245	0.86588
1581.364	0.72969	0.75583	0.78197	0.80712	0.84433	0.86778
1581.484	0.71835	0.74555	0.77275	0.7987	0.83723	0.86094
1581.603	0.71562	0.74043	0.76524	0.79467	0.83239	0.85602
1581.723	0.71143	0.73746	0.76349	0.79138	0.82793	0.84885
1581.843	0.70927	0.73273	0.75619	0.78526	0.82571	0.84584
1581.963	0.7058	0.73116	0.75652	0.78631	0.82608	0.84546
1582.083	0.69731	0.72489	0.75247	0.78126	0.82164	0.84208
1582.203	0.69655	0.72241	0.74827	0.77953	0.8198	0.84096
1582.323	0.68691	0.71434	0.74177	0.77266	0.81134	0.83348
1582.443	0.69047	0.71571	0.74095	0.76961	0.8106	0.83199
1582.562	0.68405	0.71078	0.73751	0.76649	0.80621	0.82901
1582.682	0.68787	0.71114	0.73441	0.76533	0.80511	0.82641
1582.802	0.67707	0.70431	0.73155	0.75804	0.79818	0.81863
1582.922	0.68058	0.70395	0.72732	0.75616	0.79782	0.81494
1583.042	0.66695	0.69543	0.72391	0.75247	0.79346	0.80942
1583.162	0.66529	0.69318	0.72107	0.74915	0.7902	0.80722
1583.282	0.65903	0.68993	0.72083	0.7474	0.7902	0.80649
1583.402	0.65319	0.68382	0.71445	0.74397	0.78767	0.80282
1583.521	0.6524	0.68149	0.71058	0.74073	0.78262	0.80026
1583.641	0.64782	0.67595	0.70408	0.73456	0.77579	0.79625
1583.761	0.64309	0.67123	0.69937	0.72878	0.77399	0.7908
1583.881	0.63588	0.66555	0.69522	0.72337	0.76756	0.78319
1584.001	0.63381	0.66165	0.68949	0.71975	0.76399	0.7803
1584.121	0.62773	0.65671	0.68569	0.71568	0.76186	0.77814
1584.241	0.61853	0.64836	0.67819	0.71037	0.7537	0.76916
1584.361	0.60992	0.64154	0.67316	0.70439	0.74735	0.76415
1584.48	0.60254	0.63362	0.6647	0.69597	0.73962	0.75845
1584.6	0.6022	0.63189	0.66158	0.69422	0.73682	0.75596
1584.72	0.59431	0.62592	0.65753	0.69039	0.73333	0.75135
1584.84	0.59387	0.62376	0.65365	0.68749	0.72845	0.74711
1584.96	0.58845	0.61765	0.64685	0.6809	0.72533	0.73972
1585.08	0.57725	0.60943	0.64161	0.6729	0.71771	0.73445
1585.2	0.57635	0.60687	0.63739	0.67233	0.71668	0.72921
1585.32	0.56972	0.60321	0.6367	0.66935	0.71461	0.72851
1585.44	0.56321	0.59888	0.63455	0.66597	0.71288	0.72607
1585.559	0.55866	0.59237	0.62608	0.65811	0.70533	0.71913
1585.679	0.55165	0.58613	0.62061	0.65182	0.69952	0.71255
1585.799	0.54801	0.57951	0.61101	0.64668	0.69441	0.70636
1585.919	0.54668	0.57817	0.60966	0.64392	0.69339	0.70567
1586.039	0.53969	0.57366	0.60763	0.6433	0.69237	0.70361
1586.159	0.53413	0.56668	0.59923	0.6326	0.68324	0.69642
1586.279	0.52738	0.55932	0.59126	0.62641	0.6755	0.68791
1586.399	0.52211	0.5515	0.58089	0.61809	0.6678	0.6781
1586.518	0.51973	0.55068	0.58163	0.61975	0.6678	0.67911
1586.638	0.50932	0.54503	0.58074	0.61749	0.66513	0.67844
1586.758	0.50863	0.54185	0.57507	0.60999	0.65881	0.67339
1586.878	0.49881	0.53291	0.56701	0.60341	0.65186	0.66401
1586.998	0.49703	0.52976	0.56249	0.59789	0.64922	0.65901
1587.118	0.4939	0.52645	0.559	0.59659	0.64363	0.65735
1587.238	0.48947	0.52242	0.55537	0.59169	0.6397	0.65138
1587.358	0.48467	0.51897	0.55327	0.58996	0.64035	0.65138
1587.478	0.47755	0.51425	0.55095	0.58558	0.63676	0.64675
1587.597	0.47758	0.51113	0.54468	0.58067	0.63186	0.64312
1587.717	0.47239	0.50477	0.53715	0.57529	0.62602	0.63557
1587.837	0.47051	0.5039	0.53729	0.5732	0.62342	0.63427
1587.957	0.47251	0.50358	0.53465	0.57208	0.62213	0.631
1588.077	0.46462	0.49607	0.52752	0.56647	0.61568	0.62709
1588.197	0.45914	0.48922	0.5193	0.55914	0.60894	0.62059
1588.317	0.45084	0.48148	0.51212	0.54905	0.60095	0.61381
1588.437	0.45141	0.48015	0.50889	0.54784	0.59809	0.60995
1588.556	0.44547	0.47564	0.50581	0.54353	0.59587	0.60834
1588.676	0.43471	0.46712	0.49953	0.53934	0.58764	0.5997
1588.796	0.4273	0.45842	0.48954	0.5309	0.57978	0.59269
1588.916	0.42047	0.45245	0.48443	0.52407	0.5729	0.58478
1589.036	0.41625	0.44773	0.47921	0.51927	0.56885	0.57974
1589.156	0.41291	0.4437	0.47449	0.51377	0.56419	0.5766
1589.276	0.4109	0.44075	0.4706	0.51116	0.55986	0.57096
1589.396	0.40296	0.43365	0.46434	0.50269	0.554	0.56721
1589.516	0.39574	0.42757	0.4594	0.49843	0.5494	0.56069
1589.635	0.39227	0.42196	0.45165	0.48883	0.54389	0.55357
1589.755	0.38384	0.4163	0.44876	0.48913	0.53993	0.55111
1589.875	0.38351	0.41296	0.44241	0.48185	0.53567	0.54558
1589.995	0.38148	0.41015	0.43882	0.47826	0.53052	0.54435
1590.115	0.37601	0.4033	0.43059	0.47168	0.5251	0.53946
1590.235	0.36726	0.39458	0.4219	0.46322	0.5164	0.53154
1590.355	0.36501	0.3926	0.42019	0.46061	0.5152	0.52821
1590.474	0.36299	0.3894	0.41581	0.4554	0.50984	0.52368
1590.594	0.35732	0.38679	0.41626	0.45618	0.50924	0.52247
1590.714	0.34983	0.3807	0.41157	0.45002	0.50242	0.51646
1590.834	0.34006	0.37156	0.40306	0.44305	0.49329	0.50809
1590.954	0.33374	0.36498	0.39622	0.43564	0.48655	0.50066
1591.074	0.33253	0.36306	0.39359	0.43164	0.48363	0.49357
1591.194	0.33218	0.36128	0.39038	0.43068	0.48188	0.49475
1591.314	0.33136	0.35972	0.38808	0.42742	0.47695	0.49062
1591.434	0.31875	0.35045	0.38215	0.41886	0.47289	0.48417
1591.553	0.31471	0.34314	0.37157	0.41003	0.46196	0.47427
1591.673	0.31183	0.34006	0.36829	0.40951	0.45967	0.47166
1591.793	0.30852	0.33671	0.3649	0.40493	0.45596	0.46674
1591.913	0.30491	0.33337	0.36183	0.39944	0.4534	0.4653
1592.033	0.29943	0.32782	0.35621	0.39359	0.44489	0.46041
1592.153	0.29143	0.31886	0.34629	0.38429	0.4387	0.4544
1592.273	0.29393	0.31749	0.34105	0.3799	0.43449	0.44927
1592.393	0.29109	0.31585	0.34061	0.38286	0.43421	0.4487
1592.512	0.2936	0.31797	0.34234	0.38004	0.43281	0.44728
1592.632	0.28931	0.31352	0.33773	0.37727	0.42723	0.44331
1592.752	0.28369	0.30704	0.33039	0.36993	0.42361	0.43597
1592.872	0.27234	0.29884	0.32534	0.36313	0.41476	0.42783
1592.992	0.2732	0.29851	0.32382	0.36109	0.41283	0.42449
1593.112	0.26826	0.29601	0.32376	0.36004	0.41091	0.42142
1593.232	0.26865	0.2946	0.32055	0.35601	0.40734	0.42059
1593.352	0.26116	0.28802	0.31488	0.35141	0.40133	0.41504
1593.472	0.25245	0.27863	0.30481	0.34292	0.39346	0.40842
1593.591	0.24561	0.27393	0.30225	0.33717	0.38887	0.40294
1593.711	0.24993	0.27327	0.29661	0.33406	0.38376	0.3972
1593.831	0.24602	0.26951	0.293	0.32905	0.37974	0.39529
1593.951	0.23659	0.26144	0.28629	0.3217	0.37068	0.38959
1594.071	0.23407	0.25492	0.27577	0.3136	0.363	0.37962
1594.191	0.22427	0.24683	0.26939	0.3047	0.35538	0.37213
1594.311	0.22383	0.2458	0.26777	0.29849	0.3512	0.3692
1594.431	0.21726	0.23943	0.2616	0.29888	0.34444	0.36442
1594.55	0.21433	0.23413	0.25393	0.29086	0.34055	0.35781
1594.67	0.21065	0.22993	0.24921	0.28643	0.33643	0.35334
1594.79	0.20236	0.22258	0.2428	0.2801	0.32566	0.34472
1594.91	0.20005	0.21892	0.23779	0.27559	0.32311	0.34134
1595.03	0.19201	0.21428	0.23655	0.27449	0.31804	0.33693
1595.15	0.18687	0.21115	0.23543	0.26966	0.31551	0.33409
1595.27	0.18641	0.20815	0.22989	0.26477	0.30946	0.32869
1595.39	0.17714	0.19984	0.22254	0.25745	0.30519	0.32433
1595.51	0.17003	0.19374	0.21745	0.25142	0.29746	0.31643
1595.629	0.16737	0.18963	0.21189	0.24534	0.29374	0.3091
1595.749	0.17025	0.18981	0.20937	0.24337	0.28929	0.30733
1595.869	0.16465	0.18559	0.20653	0.23945	0.28757	0.30557
1595.989	0.15777	0.17975	0.20173	0.23421	0.27971	0.30181
1596.109	0.15917	0.17733	0.19549	0.22814	0.27362	0.29556
1596.229	0.15205	0.17075	0.18945	0.22328	0.26708	0.28737
1596.349	0.1483	0.16799	0.18768	0.22051	0.2649	0.28416
1596.469	0.15142	0.17009	0.18876	0.21971	0.26515	0.2817
1596.588	0.14725	0.16559	0.18393	0.21453	0.25961	0.27851
1596.708	0.1398	0.15856	0.17732	0.20968	0.25221	0.27167
1596.828	0.13635	0.15387	0.17139	0.20313	0.24603	0.2656
1596.948	0.13061	0.15068	0.17075	0.20191	0.24461	0.26245
1597.068	0.13024	0.14968	0.16912	0.20022	0.24391	0.26149
1597.188	0.13361	0.15015	0.16669	0.19991	0.24037	0.25836
1597.308	0.12101	0.14322	0.16543	0.19396	0.23497	0.25523
1597.428	0.123	0.14029	0.15758	0.18968	0.23123	0.24854
1597.547	0.11168	0.13325	0.15482	0.18275	0.22588	0.24118
1597.667	0.11353	0.13169	0.14985	0.17821	0.22101	0.23858
1597.787	0.10959	0.12799	0.14639	0.17606	0.21802	0.23434
1597.907	0.11296	0.12759	0.14222	0.17098	0.21526	0.22989
1598.027	0.10872	0.12511	0.1415	0.16767	0.20908	0.22779
1598.147	0.10307	0.11835	0.13363	0.16096	0.20452	0.22057
1598.267	0.09768	0.1139	0.13012	0.15318	0.19796	0.21387
1598.387	0.09588	0.11242	0.12896	0.15435	0.19615	0.21526
1598.506	0.0978	0.11447	0.13114	0.15487	0.19638	0.21364
1598.626	0.09874	0.11418	0.12962	0.15431	0.19368	0.21203
1598.746	0.09054	0.10727	0.124	0.14413	0.18585	0.20243
1598.866	0.09031	0.10349	0.11667	0.14142	0.17896	0.19698
1598.986	0.08617	0.10107	0.11597	0.14209	0.17786	0.19291
1599.106	0.08495	0.10214	0.11933	0.1412	0.17543	0.19359
1599.226	0.08423	0.0999	0.11557	0.13917	0.17323	0.19133
1599.346	0.08195	0.09659	0.11123	0.136	0.17081	0.18729
1599.466	0.07659	0.09163	0.10667	0.12682	0.16208	0.18237
1599.585	0.07036	0.08698	0.1036	0.12351	0.16099	0.17726
1599.705	0.07307	0.08698	0.10089	0.12151	0.15601	0.1746
1599.825	0.07467	0.08864	0.10261	0.12187	0.15774	0.17526
1599.945	0.07456	0.0862	0.09784	0.11894	0.15213	0.17018
1600.065	0.06658	0.08091	0.09524	0.11376	0.15062	0.16535
1600.185	0.06177	0.07609	0.09041	0.10599	0.14293	0.16011
1600.305	0.06521	0.07565	0.08609	0.10503	0.13762	0.15924
1600.425	0.06803	0.0791	0.09017	0.1076	0.13741	0.1575
1600.544	0.06521	0.07713	0.08905	0.10692	0.13698	0.1575
1600.664	0.0621	0.07412	0.08614	0.10265	0.13192	0.15533
1600.784	0.06797	0.07139	0.07481	0.09294	0.12563	0.14712
1600.904	0.0595	0.06857	0.07764	0.09266	0.12354	0.14326
1601.024	0.05365	0.06397	0.07429	0.09003	0.12146	0.13559
1601.144	0.06275	0.06797	0.07319	0.09182	0.12125	0.13963
1601.264	0.05572	0.06407	0.07242	0.08996	0.11876	0.13517
1601.384	0.05036	0.05735	0.06434	0.08107	0.1105	0.12609
1601.504	0.03907	0.05078	0.06249	0.07668	0.10415	0.12399
1601.623	0.03699	0.04642	0.05585	0.07262	0.09744	0.12064
1601.743	0.04603	0.05094	0.05585	0.06936	0.09744	0.12043
1601.863	0.03358	0.04483	0.05608	0.06834	0.09906	0.11606
1601.983	0.03574	0.04404	0.05234	0.06646	0.09542	0.11233
1602.103	0.03452	0.03982	0.04512	0.06017	0.08717	0.10758
1602.223	0.03154	0.0362	0.04086	0.05541	0.08278	0.09878
1602.343	0.01942	0.03176	0.0441	0.05356	0.08059	0.10021
1602.463	0.03481	0.03756	0.04031	0.05504	0.07921	0.09898
1602.582	0.02745	0.03395	0.04045	0.05147	0.07624	0.09248
1602.702	0.02854	0.03155	0.03456	0.05134	0.06954	0.09026
1602.822	0.0235	0.02832	0.03314	0.04323	0.06778	0.08423
1602.942	0.0235	0.02832	0.03314	0.04456	0.067	0.08323
1603.062	0.02442	0.03009	0.03576	0.04709	0.0668	0.07943
1603.182	0.02383	0.0278	0.03177	0.0439	0.06388	0.08003
1603.302	0.02276	0.02485	0.02694	0.04363	0.0598	0.07645
1603.422	0.01307	0.01799	0.02291	0.03497	0.05535	0.07149
1603.542	0.0225	0.0202	0.0179	0.02864	0.05304	0.06618
1603.661	0.01551	0.01702	0.01853	0.02854	0.04882	0.05992
1603.781	0.01964	0.0201	0.02056	0.0305	0.05208	0.0607
1603.901	0.00754	0.01328	0.01902	0.0306	0.0492	0.06226
1604.021	0.01217	0.01344	0.01471	0.02463	0.04672	0.05236
1604.141	0.00703	0.01022	0.01341	0.0197	0.03912	0.05255
1604.261	5.90 × 10^−4^	0.00738	0.01417	0.01474	0.04158	0.05081
1604.381	0.00491	0.01109	0.01727	0.01706	0.03855	0.05274
1604.5	0.00188	0.00814	0.0144	0.01796	0.03893	0.04908
1604.62	0.00543	0.00819	0.01095	0.01793	0.03912	0.0439
